# Impact of lockdown due to the COVID-19 pandemic on mental health among the Libyan population

**DOI:** 10.1371/journal.pone.0267426

**Published:** 2022-04-28

**Authors:** Muhammed Elhadi, Ahmed Msherghi, Ahmed Khaled, Ahmed Alsoufi, Abdulmueti Alhadi, Asraa Kareem, Aimen Ashini, Tahani Alsharif, Alarabi Alhodiri, Emtenan Altaeb, Mona Hamed, Ahmed Itrunbah, Soha Mohmmed, Hind Alameen, Hanadi Idheiraj, Anshirah Shuwayyah, Sara Alhudhairy, Arowa Alansari, Wisam Abraheem, Hend Akl, Taha Nagib, Ayman Almugaddami, Basheer Aljameel, Siba Muamr, Suhir Alsuwiyah, Ateka Alsghair, Enas Soula, Anis Buzreg, Fatma Alagelli, Abdulfatah Aldireewi, Ahmed Bareem, Entisar Alshareea, Asmhan Gemberlo, Ahmed Zaid

**Affiliations:** 1 Faculty of Medicine, University of Tripoli, Tripoli, Libya; 2 Faculty of Medicine, University of Zawia, Az Zāwīyah, Libya; 3 Faculty of Medicine, University of Gharyan, Gharyan, Libya; 4 Faculty of Medicine, Al-Arab Medical University, Benghazi, Libya; 5 Faculty of Medicine, University of Sebha, Sebha, Libya; 6 Faculty of Medicine, Omar Al-Mukhtar University, Albayda, Libya; 7 Faculty of Medicine, University of Al-Mergib, Al Khums, Libya; Jahangirnagar University, BANGLADESH

## Abstract

**Background:**

The coronavirus disease 2019 (COVID-19) pandemic may have a potentially serious effect on mental health and increase the risk of anxiety, depression, and post-traumatic stress disorders in people. In this study, we aimed to determine the prevalence of psychological illness and the impact of the COVID-19 pandemic on the Libyan population’s mental health.

**Method:**

A cross-sectional survey, conducted in both online and paper modes and consisting of five sections, was completed in more than 30 cities and towns across Libya. The first section consisted of questions on basic demographic characteristics. The second section contained a survey related to the lockdown status, activities, related stress levels, and quarantine. The third section comprised the self-administered 9-item Patient Health Questionnaire (PHQ-9). The fourth section contained the 7-item Generalized Anxiety Disorder Scale (GAD-7), and the fifth section contained the Impact of Event Scale-Revised (IES-R).

**Result:**

Of the 31,557 respondents, 4,280 (13.6%) reported severe depressive symptoms, with a mean [standard deviation (SD)] PHQ-9 score of 8.32 (5.44); 1,767 (5.6%) reported severe anxiety symptoms, with a mean (SD) GAD-7 score of 6 (4.6); and 6,245 (19.8%) of the respondents reported post-traumatic stress disorder (PTSD), with a mean (SD) score of 15.3 (18.85). In multivariate analysis, young age, being female, unmarried, educated, or victims of domestic violence or abuse, work suspension during the pandemic, and having increased workload, financial issues, suicidal thoughts, or a family member with or hospitalized due to COVID-19 were significantly associated with a high likelihood of depressive and anxiety symptoms, as well as PTSD. Internal displacement due to civil war was also associated with PTSD.

**Conclusion:**

To our knowledge, this is the first study to analyze the psychological impacts of the COVID-19 pandemic and civil war in Libya. Further study on the development of strategies and interventions aimed at reducing the mental disease burden on the Libyan population is warranted.

## Introduction

The outbreak of coronavirus disease 2019 (COVID-19), caused by severe acute respiratory syndrome coronavirus 2 (SARS-CoV-2), began in December 2019 [1]. As of February 22, 2022, more than 426 million cases and 5.89million deaths due to COVID-19 have been recorded globally [2]. COVID-19 has been proposed as a serious event that has had a significant impact on the mental well-being of the population worldwide [3].

A limited number of studies have described the prevalence of mental health disorders associated with the COVID-19 pandemic among the global population [4, 5]. The COVID-19 pandemic may have a potentially serious effect on mental health and increase the risk of anxiety, depression, and post-traumatic disorders, especially in patients with COVID-19 or individuals who have had contact with COVID-19 patients.

Previous studies have focused on a few countries and identified several psychological stressors; however, most of these studies either focused on specific socioeconomic cultures or used small sample sizes that included specific populations such as the elderly or young age groups [4–7]. The Libyan population has been suffering from an ongoing civil war since 2011, which has affected the population’s financial and psychological well-being [8]. The civil war and conflict could have short- and long-term effects on populations, especially medically-defined post-traumatic stress disorder (PTSD) [9]. In addition, there are no training programs for psychiatrists in Libya, which has resulted in low levels of mental healthcare services. This has been further exacerbated by the fact that Libya lacks the required mental healthcare facilities, with an estimated report of only 0.2 psychiatrists per 100,000 individuals [10, 11]. Many cities in Libya lack mental healthcare services and centers, and it has been estimated that there are only two main psychiatric hospitals in Libya [12].

Regarding the current status of mental health during the ongoing COVID-19 pandemic, a study conducted in China revealed a 30% and 17% rise in the rates of anxiety and depression, respectively [5]. Another study in Italy reported 37, 17.3, and 20.8% increases in post-traumatic stress symptoms, depression, and anxiety, respectively [13]. In another study conducted in Spain, it was reported that 18.7% of individuals had depressive symptoms, 21.6% had anxiety, and 15.8% had PTSD [7].

Currently, few studies were conducted on the psychological impact of COVID-19 lockdown in the African population; additionally, the mental health status of the general Libyan population is unknown. Therefore, to our knowledge, there have been no previous studies on the psychological impact of COVID-19 lockdown on the general population of Libya.

In this context, we present the first study conducted among the Libyan population with the aim of determining the prevalence of psychological illness and the impact of the COVID-19 pandemic on the mental health of this region. The results of this study may assist policy makers and international organizations by providing the necessary population-based analysis of psychological well-being during the COVID-19 pandemic and the ongoing civil war crisis in Libya.

## Materials and methods

### Study design, setting, and period

A cross-sectional survey was conducted in more than 30 cities and towns in Libya using both online and paper modes. This study took place over a period of 12 successive days, from May 1, 2020, to May 26, 2020. The electronic survey was sent through email and social media. The paper survey was conducted anonymously at several points in each city to cover a larger geographical area with different study populations. The paper questionnaire was collected by the authors at each collection point in the Libyan cities.

### Sample size and sampling technique

According to the UN Data and Worldometers, the Libyan population in mid-2020 was estimated to be 6,871,292 people. The sample size was calculated based on a single proportion formula, considering a previous study performed in China with a sample population proportion of 53.8% [5]. Using a cross-sectional study design, where n = required sample size (n = Z (α/2) 2 pq/d2), we calculated the sample size based on the following parameters:

The Libyan population was 6,871,292, p = 0.54, q (1-p) = 0.46, 99% CI, and 1% margin of error. We estimated 16,442 as the minimum sample size required to represent the true population. A convenience sampling method was used in this study.

### Participants

The inclusion criteria were as follows: people who had lived for at least five consecutive years in Libya regardless of their nationality, those who were able to read and write, and those aged 18 years or above. Responses from those who did not fully complete the questionnaire were excluded from the study. We sent the survey via email, mobile messages, and paper to more than 40,000 participants.

The survey comprised five sections. The first section consisted of questions on basic demographic characteristics and general questions related to socioeconomic status and sleep changes. The second section contained a lockdown survey (S1 and S2 Files), with several questions related to people’s lockdown status, activities, related stress level, and quarantine. The third section comprised the self-administered 9-item Patient Health Questionnaire (PHQ-9) [14, 15] with a cutoff score of ≥15. The fourth section contained the 7-item Generalized Anxiety Disorder Scale (GAD-7) [16] with a cutoff score of ≥15, and the fifth section contained the Impact of Event Scale-Revised (IES-R) [17] with a cutoff score of ≥33. The survey was conducted anonymously. All participants provided informed consent before completing the survey.

### Data quality control

The data instruments used in the study were translated into Arabic by two independent linguistic experts. They were then translated back to English to determine internal consistency and intended meanings. Any discrepancies were resolved by consulting the authors. Psychometric tools such as PHQ-9, GAD-7, and IES-R were used in a pilot study of 30 respondents to achieve the highest possible internal consistency, as determined by a Cronbach’s alpha above 0.7. Regarding internal consistency, as determined by a Cronbach’s alpha of 0.828, PHQ-9 was found to have an internal consistency of 0.84, while GAD-7 demonstrated an internal consistency of 0.88 and ISE-R demonstrated an internal consistency of 0.86. The first three authors trained the data collectors in the week prior to the study.

### Statistical analysis

Descriptive statistical analyses were conducted in terms of frequency and percentage. The chi-square test was used to determine statistically significant associations between variables. The Mann–Whitney U test was used to determine differences between the two gender groups based on age. The Wilcoxon signed-rank test was used to determine whether there was a median difference between paired questions in the lockdown questionnaire before and during lockdown. Multinomial logistic regression was used to determine the impact of variables on mental health outcomes (depression, anxiety, and PTSD). Statistical analysis was performed using IBM’s SPSS Statistics package for Windows (Version 25.0) and Stata SE version 16.0 (StataCorp, Texas, USA).

### Ethical approval

Ethical approval was obtained from the Bioethics Committee of the Biotechnology Research Center of the Ministry of Higher Education and Scientific Research in Libya. All participants provided written informed consent prior to participating in the study, without any identifiable data.

## Results

From an estimated 40,000 who received the survey, we received valid responses from 31,557 participants, with a response rate of 78.89%. Of the 31,557 respondents, 20,755 (65.8%) were females. The median (interquartile range) of the participants was 8 (23–5), ranging from 18 to 80 years. Most of the participants were unmarried 19075 (60.4%), having higher or university level education 21131 (67%), not infected with COVID-19 30314 (96.1%), and 20670 (65.5%) reported work suspended due to the pandemic. While only 1256 (4%) were internally displaced and 14254 (45.2%) reported financial difficulties. We observed that women were having higher frequency of domestic abuse or violence. While men were reported higher frequency of suicidal ideation during the lockdown compared to women. Table 1 presents the basic characteristics of the participants, along with the differences between men and women.

**Table 1:** Basic characteristics of study participants (n = 31557).

Variables	Total (%)	Female (%)	Male (%)	p-value
	n = 31557	n = 20755	n = 10802	
**Age, median (IQR)**	28 (23–35)	28 (22–34)	29 (24–39)	<0.001**
**Age range (years)**				<0.001**
18–30	20121 (63.8)	14077 (67.8)	6044 (56)	
31–40	6650 (21.1)	4182 (20.1)	2468 (22.8)	
41–50	3127 (9.9)	1800 (8.7)	1327 (12.3)	
**>**50	1659 (5.3)	696 (3.4)	963 (8.9)	
**Marital status**				0.989
Married	12482 (39.6)	8210 (39.6)	4272 (39.5)	
Unmarried (Single, divorced, widowed, …)	19075 (60.4)	12545 (60.4)	6530 (60.5)	
**Education level**				
Elementary	378 (1.2)	168 (0.8)	210 (1.9)	<0.001**
Preparatory	1255 (4)	736 (3.5)	519 (4.8)	
Secondary	5743 (18.2)	3529 (17)	2214 (20.5)	
University education/Higher education	21131 (67)	14740 (71)	6391 (59.2)	
Postgraduate studies	3050 (9.7)	1582 (7.6)	1468 (13.6)	
**Currently internally displaced**	1256 (4)	708 (3.4)	548 (5.1)	<0.001**
**Family member / Loved one status**				<0.001**
Nobody was infected	30314 (96.1)	20055 (96.6)	10259 (95)	
Infected	870 (2.8)	506 (2.4)	364 (3.4)	
Infected and hospitalized	343 (1.1)	183 (0.9)	160 (1.5)	
Deceased	30 (0.1)	11 (0.1)	19 (0.2)	
**Work status after COVID-19 pandemic**				<0.001**
No change	5420 (17.2)	2887 (13.9)	2533 (23.4)	
Increased workload	1822 (5.8)	908 (4.4)	914 (8.5)	
Teleworking	3645 (11.6)	1919 (9.2)	1726 (16)	
Work is suspended	20670 (65.5)	15041 (72.5)	5629 (52.1)	
**Suffering from financial issues**	14254 (45.2)	9316 (44.9)	4938 (45.7)	0.161
**Domestic violence/abuse**	9959 (31.6)	7062 (34)	2897 (26.8)	<0.001**
**Considered suicide during lockdown**	2384 (7.6)	1386 (6.7)	998 (9.2)	<0.001**
**PHQ ≥15**	4280 (13.6)	3062 (14.8)	1218 (11.3)	<0.001**
**GAD ≥15**	1767 (5.6)	1279 (6.2)	488 (4.5)	<0.001**
**IES-R≥ 33**	6245 (19.8)	4115 (19.8)	2130 (19.7)	0.819

* = Significant at p < 0.05;

** = Significant at p < 0.001; PHQ: Patient Health Questionnaire; GAD: General Anxiety Disorder; IES-R: Impact of Event Scale-Revised.

### Mental health of the study population

**Fig 1:**
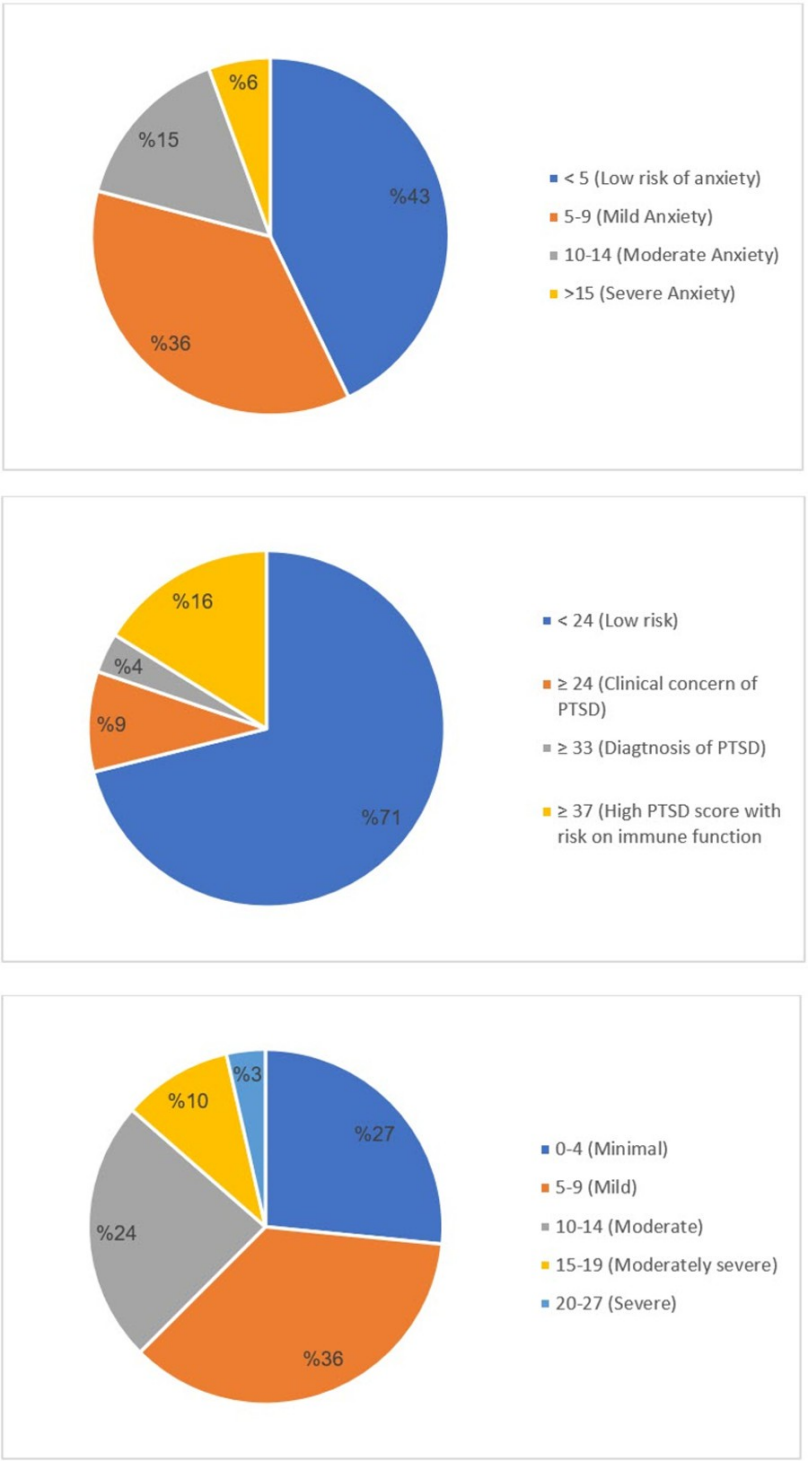
**(A)** Distribution of anxiety among study participants (n = 31,557). **(B)** Distribution of post-traumatic stress disorder among study participants (n = 31,557). **(C)** Distribution of depression among study participants (n = 31,557).

Of the 31,557 respondents, 4,280 (13.6%) reported severe depressive symptoms, with a mean (SD) PHQ-9 score of 8.32 (5.44) and a median of 8 (IQR = 8, range 0–27). Of the respondents, 1,767 (5.6%) reported severe anxiety symptoms, with a mean (SD) GAD-7 score of 6 (4.6) and a median score of 5 (IQR = 5, range 0–21). In addition, 6,245 (19.8%) of the respondents reported PTSD using the ISE-R scale, with a mean (SD) score of 15.3 (18.85) and a median IES-R score of 6 (IQR = 27, range 0–88). There was a significant association between gender and severe depressive symptoms and anxiety (χ2(1) = 73.280, p ≤ 0.001 for depression; χ2(1) = 36.57, p < 0.001). However, there was no significant association between gender and PTSD binary levels. The distribution of different degrees of anxiety, PTSD, and depression based on their scores are shown in Fig 1A–1C.

### Impact of the lockdown due to COVID-19

Table 2 presents the answers to the COVID-19 lockdown questionnaire. We observed that approximately 27,183 (86.1%) individuals followed the isolation procedures according to governmental guidance. Moreover, 6,161 (19.5%) individuals reported that they adhered to social distancing norms and never violated them; 6,838 (21.7%) reported that they adhered to the social distancing norms most of the time, and 11,811 (37.4%) reported that they adhered to the guidelines of social distancing as much as possible. Of the respondents, 19,649 (62.3%) individuals thought that lockdown is a good idea to reduce viral transmission. In addition, most of the participants reported that they felt more relaxed after performing sports, doing household chores, talking to somebody, avoiding annoying persons, performing work and duties, and eating with their families.

**Table 2:** Survey on impact of lockdown implemented owing to COVID-19.

Questions	Yes (%)	No (%)
*1*. *Occupational and general setting*		
1.1 Did you isolate yourself?		
I isolated myself according to the instructions of the Government Authorities	27183 (86.1)	
I isolated myself because I tested positive for COVID or was in contact with a confirmed case	335 (1.1)	
I am currently hospitalized as a result of infection	224 (0.7)	
I do not practice any kind of personal isolation	3815 (12.1)	
1.2 Before the start of the lockdown, how many hours did you spend (approximately) outside your home or residence every day?		
0–1 hour	5472 (17.3)	
2–3 hours	5426 (17.2)	
4–5 hours	7837 (24.8)	
6–8 hours	7728 (24.5)	
More than 8 hours	5094 (16.1)	
1.3 During the lockdown period, how many hours did you spend (approximately) outside your home or residence every day?		
0–1 hour	19127 (60.6)	
2–3 hours	5402 (17.1)	
4–5 hours	3355 (10.6)	
6–8 hours	2043 (6.5)	
More than 8 hours	1630 (5.2)	
1.4 Source through which you received/receive information regarding spread of COVID-19 pandemic (can chose more than one option)		
Governmental authorities	23054 (73.1)	
Media	22645 (71.8)	
Friends and relatives	15416 (48.9)	
Social media	23702 (75.1)	
1.5 How was your adherence to the lockdown instructions and safe distancing imposed by government authorities to fight COVID-19?		
No adherence	2290 (7.3)	
Little adherence	4457 (14.1)	
Adhered as much as possible	11811 (37.4)	
Was adherent most of the time	6838 (21.7)	
Never violated the instructions	6161 (19.5)	
2. Lockdown related activities		
2.1 During the lockdown period, I feel better after doing the following (more than one answer can be chosen)		
Physical activity and exercise	16355 (51.8)	
Watching movies and series	19111 (60.6)	
Following the news	7128 (22.6)	
Carrying out some tasks or assignments	21133 (67)	
Doing housework	19555 (62)	
Talking to someone	22987 (72.8)	
Staying away from annoying people whom I spend the lockdown with	21153 (67)	
Eating with my family	24911 (78.9)	
2.2 During the lockdown period, did you contact any person who does not reside in the same residence as you in person?	17402 (55.1)	14155 (44.9)
2.3 During lockdown, did you communicate with anyone not living with you through a phone call/voice call over internet?	26464 (83.9)	5093 (16.1)
3. Attitude and personal behavior		
3.1 Do you think quarantine is a good idea?	19649 (62.3)	11908 (37.7)
3.2 What was your stress level before the lockdown was implemented?		
I was calm	15817 (50.1)	
I was neither calm nor anxious	9145 (29)	
I was anxious	4402 (13.9)	
I was very anxious	2193 (6.9)	
3.3 What is your stress level during lockdown?		
I am calm	13562 (43)	
I am neither calm nor anxious	8638 (27.4)	
I am anxious	6940 (22)	
I am very anxious	2417 (7.7)	
3.4 Did you suffer from depressive episodes during lockdown?	14307 (45.3)	17250(54.7)
3.5 Did you suffer from anxiety and/or stress episodes during lockdown?	23150 (73.4)	8407 (26.6)
3.6 Did you suffer from domestic violence?	9959 (31.6)	21598 (68.4)
3.7 Did you suffer from financial issues during lockdown?	14254 (45.2)	17303 (54.8)
3.8 Did you seriously consider committing suicide during lockdown?	2384 (7.6)	29173 (92.4)
3.9 Did you encounter problems surrendering to sleep or staying asleep?	15913 (50.4)	15644 (49.6)
3.10 Have you tried to eliminate anxiety by resorting to smoking cigarettes, using alcohol, or drugs?	3526 (11.2)	28031 (88.8)

Questions 1.2 and 1.3 were about the average number of hours spent outside home. Among those who answered the survey, we observed a change in the number of hours spent outside before and during lockdown. The Wilcoxon signed-rank test was used to determine the effect of lockdown on hours spent outside home before and during lockdown. There was a significant difference in the median number of hours spent outside before and during lockdown (z = 115.84, p < 0.001).

Questions 3.2 and 3.3 were based on the stress levels before and during the lockdown. The results of the Wilcoxon signed-rank test indicated that there was a significant increase in the median stress level during lockdown compared to that before lockdown (z = -25.215, p < 0.001).

Regarding sleep changes, 15,913 (50.4%) individuals reported experiencing either sleep disturbances or excessive sleep. In addition, 3,526 (11.2%) individuals reported trying to reduce anxiety and stress by using drugs, smoking tobacco, or drinking alcohol.

### Analysis of depressive symptoms (PHQ-9≥15)

Bivariate and multivariate binomial logistic regression analyses were performed to analyze the study variables and their association with depressive symptoms (PHQ-9 ≥15) compared with non-depressive symptoms (PHQ-9<15). In bivariate analysis, there was a significant association between depressive symptoms and gender, marital status, education level, internal displacement due to civil war, work status during the pandemic, infectious status, having a family member or loved one infected with COVID-19, financial issues, domestic violence, and suicidal ideation (p < 0.001). The Mann–Whitney U test was used to determine whether there were differences in the age of study participants between the depressive and non-depressive symptom groups. Median age was significantly lower in patients with depression (25 years, IQR [21–28]) than in those without depression (28 years, IQR (23–36)), p < 0.001. Table 3 presents the results of the bivariate and multivariate analysis.

**Table 3:** Bivariate and multivariate binomial logistic regression analysis of depressive symptoms (PHQ-9 ≥15).

Variable	Total n (%)	PHQ-9 (<15)	PHQ-9≥15	*χ2*	p-value	Odds ration	95% CI for Odds ration		P-value
							Lower	Upper	
**Age (years), Median (IQR)**	28 (23–35)	28 (23–36)	25 (21–28)		<0.001**	0.97	0.96	0.98	<0.001**
**Gender**									
• Male	10802 (34.2)	9584 (35.1)	1218 (28.5)	73.28	<0.001**	1 (ref)			
• Female	20755 (65.8)	17693 (64.9)	3062 (71.5)			1.22	1.13	1.32	<0.001**
**Marital status**									
• Unmarried (Single, divorced, widowed, …)	19075 (60.4)	15803 (57.9)	3272 (76.4)	530.34	<0.001**	1.63	1.49	1.78	<0.001**
• Married	12482 (39.6)	11474 (42.1)	1008 (23.6)			1 (ref)			
**Highest education**									
• Elementary	378 (1.2)	344 (1.3)	34 (0.8)	110.44	<0.001**	1 (ref)			
• Preparatory	1255 (4)	1074 (3.9)	181 (4.2)			2.17	1.42	3.33	<0.001**
• Secondary	5743 (18.2)	4768 (17.5)	975 (22.8)			2.49	1.66	3.72	<0.001**
• University education / Higher education	21131 (67)	18329 (67.2)	2802 (65.5)			2.25	1.51	3.35	<0.001**
• Postgraduate studies	3050 (9.7)	2762 (10.1)	288 (6.7)			2.21	1.46	3.34	<0.001**
**Currently internally displaced**									
• Yes	1256 (4)	1023 (3.8)	233 (5.4)	27.76	<0.001**	1.17	.99	1.39	0.059
• No	30301 (96)	26254 (96.2)	4047 (94.6)			1 (ref)			
**Work status during COVID-19 pandemic**									
• No change in working	5420 (17.2)	4903 (18)	517 (12.1)	109.57	<0.001**	1 (ref)			
• Increased workload	1822 (5.8)	1532 (5.6)	290 (6.8)			1.55	1.31	1.84	<0.001**
• Teleworking	3645 (11.6)	3199 (11.7)	446 (10.4)			1.47	1.27	1.71	<0.001**
• Work is suspended	20670 (65.5)	17643 (64.7)	3027 (70.7)			1.69	1.51	1.89	<0.001**
**COVID-19 infectious status**									
Not infected, no contact	30663 (97.2)	26627 (97.6)	4036 (94.3)		<0.001**	1 (ref)			
Not infected, infected contact	533 (1.7)	406 (1.5)	127 (3)	163.527		1.02	0.78	1.32	0.87
Infected, not hospitalized	265 (0.8)	175 (0.6)	90 (2.1)			1.67	1.22	2.29	0.001*
Infected and hospitalized	96 (0.3)	69 (0.3)	27 (0.6)			1.24	0.74	2.08	0.39
**Family member / Loved one status**									
• Nobody was infected	30314 (96.1)	26388 (96.7)	3926 (91.7)			1 (ref)			
• Infected	870 (2.8)	628 (2.3)	242 (5.7)	254.44	<0.001**	2.31	1.91	2.79	<0.001**
• Infected and hospitalized	343 (1.1)	235 (0.9)	108 (2.5)			2.33	1.75	3.10	<0.001**
• Deceased	30 (0.1)	26 (0.1)	4 (0.1)			1.42	0.47	4.33	0.53
**Suffering from financial issues**									
• Yes	14254 (45.2)	11603 (42.5)	2651 (61.9)	562.28	<0.001**	1.69	1.57	1.82	<0.001**
• No	17303 (54.8)	15674 (57.5)	1629 (38.1)			1 (ref)			
**Domestic violence / abuse**									
• Yes	9959 (31.6)	7335 (26.9)	2624 (61.3)			3.00	2.79	3.23	<0.001**
• No	21598 (68.4)	19942 (73.1)	1656 (38.7)	2028.93	<0.001**	1 (ref)			
**Considered suicide during lockdown**									
• Yes	2384 (7.6)	1393 (5.1)	991 (23.2)	1725.34	<0.001**	3.69	3.33	4.08	<0.001**
• No	29173 (92.4)	25884 (94.9)	3289 (76.80)			1 (ref)			

IQR, interquartile range;

*, significant at p < 0.05;

**, significant at p < 0.001; PHQ-9, Patient Health Questionnaire.

Multivariate binomial logistic regression was performed to determine the effects of the combination of study characteristics on the likelihood of participants having depressive symptoms (PHQ-9 ≥15). We found that younger age was associated with a higher likelihood of depressive symptoms (OR = 0.97 [0.96, 0.98]). Females had 1.22 times higher odds of exhibiting depressive symptoms than males (OR = 1.22 [1.13, 1.32]). Additionally, unmarried people had 1.63 times higher odds of being depressed than married people (OR = 1.63 [1.49, 1.78]). Higher education level was significantly associated with higher odds of being depressed, and those with secondary- or university-level education had the highest likelihood of being depressed (OR = 2.49 [1.66, 3.72]), OR = 2.25 [1.51, 3.35], respectively). Individuals whose work was suspended owing to the COVID-19 pandemic or those who turned to teleworking had a higher likelihood of being depressed (OR = 1.69 [1.51, 1.89], OR = 1.47 [1.27, 1.71], respectively). Individuals with a previous history of infection with COVID-19 without hospitalization had 1.67 times higher odds of being depressed than those who were not infected (OR = 1.67 [1.22, 2.29]). Having a relative or loved one infected with COVID-19 was significantly associated with depressive symptoms. Individuals who had financial issues during the pandemic, were exposed to domestic abuse or violence, or had suicidal ideation were more likely to have depressive symptoms (OR = 1.69 [1.57, 1.82], OR = 3 [2.79, 3.23], OR = 3.69 [3.33, 4.08], respectively). Although internal displacement due to civil war was significantly associated with depressive symptoms in the bivariate analysis, it was not significantly associated with a higher likelihood of depressive symptoms in the multivariate analysis. Table 3 provides a detailed overview of the multivariate binomial logistic regression model.

### Analysis of anxiety symptoms (GAD-7 ≥15)

Bivariate and multivariate binomial logistic regression analyses were performed to analyze study variables and their association with anxiety symptoms (GAD-7 ≥15) compared with non-anxiety symptoms (GAD-7 <15), as determined using the GAD-7 scale. The Mann–Whitney U test was performed to determine whether there were differences in the age of the study participants between the two groups. Median age was significantly lower in people with anxiety symptoms (25 years, IQR [21–29]) than in participants without anxiety symptoms (28 years, IQR [23–35]) (p < 0.001). In bivariate analysis, there was a significant association between severe anxiety symptoms and gender, marital status, education level, internal displacement due to civil war, work status, COVID-19 infection status, family members or loved ones being infected with COVID-19, financial issues, domestic violence, and suicidal ideation (p < 0.05). Table 4 presents the results of the bivariate and multivariate analysis.

**Table 4:** Bivariate and multivariate binomial logistic regression analysis of severe anxiety symptoms (GAD-7 ≥15).

Variable	Total n (%)	GAD-7 (<15)	GAD-7 ≥ 15	*χ2*	p-value	Odds ration	95% CI for Odds ration		p-value
							Lower	Upper	
**Age (years), Median (IQR)**	28 (23–35)	28 (23–35)	25 (21–29)		<0.001**	0.978	0.971	0.985	<0.001**
**Gender**									
• Male	10802 (34.2)	10314 (34.6)	488 (27.6)	36.35	<0.001**	1 (ref)			
• Female	20755 (65.8)	19476 (65.4)	1279 (72.4)			1.17	1.04	1.31	0.007*
**Marital status**									
• Unmarried (Single, divorced, widowed, …)	19075 (60.4)	12028 (40.4)	454 (25.7)	150.41	<0.001**	1.31	1.15	1.49	<0.001**
• Married	12482 (39.6)	17762 (59.6)	1313 (74.3)			1 (ref)			
**Highest education**									
• Elementary	378 (1.2)	366 (1.2)	12 (0.7)	72.43	<0.001**	1 (ref)			
• Preparatory	1255 (4)	1176 (3.9)	79 (4.5)			2.31	1.21	4.42	0.01*
• Secondary	5743 (18.2)	5308 (17.8)	435 (24.6)			2.54	1.37	4.69	0.003*
• University education / Higher education	21131 (67)	20001 (67.1)	1130 (64)			2.06	1.12	3.79	0.02*
• Postgraduate studies	3050 (9.7)	2939 (9.9)	111 (6.3)			1.98	1.05	3.74	0.03*
**Currently internally displaced**									
• Yes	1256 (4)	1166 (3.9)	90 (5.1)	6.07	0.014*	1.16	0.91	1.48	0.215
• No	30301 (96)	28624 (96.1)	1677 (94.9)			1 (ref)			
**Work status during COVID-19 pandemic**									
• No change in working	5420 (17.2)	5267 (17.7)	153 (8.7)	145.48	<0.001**	1 (ref)			
• Increased workload	1822 (5.8)	1703 (5.7)	119 (6.7)			2.11	1.63	2.75	<0.001**
• Teleworking	3645 (11.6)	3511 (11.8)	134 (7.6)			1.44	1.12	1.84	0.004*
• Work is suspended	20670 (65.5)	19309 (64.8)	1361 (77)			2.42	2.02	2.90	<0.001**
**COVID-19 infectious status**									
Not infected, no contact	30663 (97.2)	28979 (97.3)	1684 (95.3)	29.53	<0.001**	1 (ref)			
Not infected, infected contact	533 (1.7)	491 (1.6)	42 (2.4)			1.05	0.71	1.56	0.77
Infected, not hospitalized	265 (0.8)	233 (0.8)	32 (1.8)			1.56	1.00	2.44	0.05
Infected and hospitalized	96 (0.3)	87 (0.3)	9 (0.5)			1.21	0.57	2.55	0.62
**Family member / Loved one status**									
• Nobody was infected	30314 (96.1)	28641 (96.1)	1673 (94.7)	18.76	<0.001**	1 (ref)			
• Infected	870 (2.8)	815 (2.7)	55 (3.1)			0.92	0.66	1.29	0.65
• Infected and hospitalized	343 (1.1)	306 (1)	37 (2.1)			1.41	0.93	2.14	0.1
• Deceased	30 (0.1)	28 (0.1)	2 (0.1)			1.66	0.37	7.41	0.5
**Suffering from financial issues**									
• Yes	14254 (45.2)	13063 (43.9)	1191 (67.4)	373.59	<0.001**	1.79	1.61	2.01	<0.001**
• No	17303 (54.8)	16727 (56.1)	576 (32.6)			1 (ref)			
**Domestic violence / abuse**									
• Yes	9959 (31.6)	8705 (29.2)	1254 (71)			3.76	3.36	4.21	<0.001**
• No	21598 (68.4)	21085 (70.8)	513 (29)	1345.9	<0.001**	1 (ref)			
**Considered suicide during lockdown**									
• Yes	2384 (7.6)	1887 (6.3)	497 (28.1)	1134.3	<0.001**	3.92	3.45	4.46	<0.001**
• No	29173 (92.4)	27903 (93.7)	1270 (71.9)			1 (ref)			

IQR, interquartile range;

* = significant at p < 0.05;

** = significant at p < 0.001; GAD: general anxiety disorder.

Multivariate binomial logistic regression was performed to determine the effects of study characteristics on the likelihood of participants having anxiety symptoms (GAD-7 ≥15) or not (GAD-7 < 15). We found that younger age was associated with an increased likelihood of experiencing anxiety symptoms. Females were 1.17 times more likely to exhibit anxiety symptoms than males (OR = 1.17 [1.04, 1.31]). In addition, unmarried individuals had a 1.31 times higher likelihood of having anxiety symptoms than married participants (OR = 1.31 [1.15, 1.49]). Similar to depressive symptoms, anxiety symptoms were higher in those with higher levels of education. Work status during the pandemic was independently associated with anxiety symptoms, especially in those whose workload increased or whose work was suspended during the pandemic (OR = 2.11 [1.63, 2.75], OR = 2.44 [2.02, 2.90], respectively). Individuals who suffered from financial issues during the pandemic, were exposed to domestic abuse or violence, or had suicidal ideation were more likely to have anxiety symptoms (OR = 1.79 [1.61, 2.01], OR = 3.76 [3.36, 4.21], OR = 3.92 [3.45, 4.46], respectively). However, internal displacement due to civil war was significantly associated with anxiety symptoms in bivariate analysis but not in multivariate analysis. Table 4 provides a detailed overview of the multivariate binomial logistic regression model.

### Analysis of PTSD (IES-R ≥33)

Bivariate and multivariate binomial logistic regression analyses were performed to analyze the study variables and their association with PTSD. This was measured using the IES-R scale, where we compared individuals with a cutoff score of IES-R ≥33 with those with a cutoff score of IES-R <33. In bivariate analysis, there was a significant association between PTSD and gender, marital status, education level, internal displacement, work status during the pandemic, COVID-19 infection status, family members or loved ones being infected with COVID-19, financial issues, domestic violence, and suicidal ideation (p < 0.001). The Mann–Whitney U test was performed to determine whether there were differences in the age of study participants with low risk of PTSD (IES-R <33) and those with a high risk of PTSD (IES-R ≥33). The median age was significantly different between those with PTSD (28 years, IQR [22–33]) and those without PTSD (28 years, IQR 23–36). Table 5 presents the results of the bivariate and multivariate analysis.

**Table 5:** Bivariate and multivariate binomial logistic regression analysis of post-traumatic stress disorder (ISE-R ≥33).

Variable	Total n (%)	IES-R< 33	IES-R≥ 33	*χ2*	p-value	Odds ration	95% CI for Odds ration		p-value
							Lower	Upper	
**Age (years), Median (IQR)**	28 (23–35)	28 (23–36)	28 (22–33)		<0.001**	0.995	0.991	0.998	0.003*
**Gender**									
• Male	10802 (34.2)	8672 (34.3)	2130 (34.1)	0.052	0.819	1 (ref)			
• Female	20755 (65.8)	16640 (65.7)	4115 (65.9)			1.07	1.005	1.143	0.034*
**Marital status**									
• Unmarried (Single, divorced, widowed, …)	19075 (60.4)	10324 (40.8)	2158 (34.6)	81.35	<0.001**	1.139	1.06	1.22	<0.001**
• Married	12482 (39.6)	14899 (59.2)	4097 (65.4)			1 (ref)			
**Highest education**									
• Elementary	378 (1.2)	297 (1.2)	81 (1.3)	69.69	<0.001**	1 (ref)			
• Preparatory	1255 (4)	946 (3.7)	309 (3.9)			1.75	1.27	2.42	0.001
• Secondary	5743 (18.2)	4436 (17.5)	1307 (20.9)			1.78	1.32	2.39	<0.001**
• University education / Higher education	21131 (67)	17198 (67.9)	3933 (63)			1.59	1.18	2.13	0.002*
• Postgraduate studies	3050 (9.7)	2435 (9.6)	615 (9.8)			1.73	1.27	2.34	<0.001**
**Currently internally displaced**									
• Yes	1256 (4)	854 (3.4)	402 (6.4)	122.99	<0.001**	1.26	1.09	1.45	0.001*
• No	30301 (96)	24458 (96.6)	5843 (93.6)			1 (ref)			
**Work status during COVID-19 pandemic**									
• No change in working	5420 (17.2)	4489 (17.7)	931 (14.9)	384.77	<0.001**	1 (ref)			
• Increased workload	1822 (5.8)	1182 (4.7)	640 (10.2)			2.07	1.82	2.36	<0.001**
• Teleworking	3645 (11.6)	2738 (10.8)	907 (14.5)			1.60	1.43	1.78	<0.001**
• Work is suspended	20670 (65.5)	16903 (66.8)	3767 (60.3)			1.13	1.04	1.23	0.004*
**COVID-19 infectious status**									
Not infected, no contact	30663 (97.2)	24965 (98.6)	5698 (91.2)	1014.36	<0.001**	1 (ref)			
Not infected, infected contact	533 (1.7)	192 (0.8)	341(5.5)			3.64	2.94	4.51	<0.001**
Infected, not hospitalized	265 (0.8)	101 (0.4)	164 (2.6)			3.00	2.25	4.004	<0.001**
Infected and hospitalized	96 (0.3)	54 (0.2)	42 (0.7)			1.45	0.92	2.28	0.102
**Family member / Loved one status**									
• Nobody was infected	30314 (96.1)	24687 (97.5)	5627 (90.1)			1 (ref)			
• Infected	870 (2.8)	500 (2)	370 (5.9)	839.02	<0.001**	1.64	1.38	1.94	<0.001**
• Infected and hospitalized	343 (1.1)	107 (0.4)	236 (3.8)			4.01	3.07	5.24	<0.001**
• Deceased	30 (0.1)	18 (0.1)	12 (0.2)			1.46	0.64	3.33	0.35
**Suffering from financial issues**									
• Yes	14254 (45.2)	10776 (42.6)	3478 (55.7)			1.51	1.42	1.60	<0.001**
• No	17303 (54.8)	14536 (57.4)	2767 (44.3)	348.14	<0.001**	1 (ref)			
**Domestic violence / abuse**									
• Yes	9959 (31.6)	6938 (27.4)	3021 (48.4)			2.01	1.89	2.14	<0.001**
**•** No	21598 (68.4)	18374 (72.6)	3224 (51.6)	1019.31	<0.001**	1 (ref)			
**Considered suicide during lockdown**									
• Yes	2384 (7.6)	1271 (5)	1113 (17.8)			2.49	2.26	2.74	<0.001**
• No	29173 (92.4)	24041 (95)	5132 (82.2)	1175.3	<0.001**	1 (ref)			

IQR, interquartile range;

* = significant at p < 0.05;

** = significant at p < 0.001; IES-R: Impact of Event Scale-Revised.

Multivariate binomial logistic regression was performed to determine the effects of study characteristics combined with the likelihood of participants having PTSD (IES-R ≥33). We found that younger age was associated with an increased likelihood of developing PTSD. Females were 1.07 times more likely to have PTSD than males (OR = 1.07 [1.005, 1.143]). Moreover, unmarried people had a 1.39 times higher likelihood of having PTSD than married subjects (OR = 1.139 [1.06, 1.22]). Similar to anxiety and depressive symptoms, PTSD was significantly associated with higher education levels. Being internally displaced due to civil war was significantly associated with 1.26 times higher odds of exhibiting PTSD (OR = 1.26, [1.09, 1.45]). Work status changes during the pandemic is statistically associated with increased likelihood of PTSD, especially in those who reported increased work load (OR = 2.07 [1.82, 2.36]). Notably, those who were infected with COVID-19 without hospitalization or those who had recent contact with infected patients were statistically associated with higher odds of having PTSD, (OR = 3 [2.25, 4.004], OR = 3.64 [2.94, 4.51], respectively).

Participants who reported having suffered from financial issues during the pandemic, exposure to domestic violence or abuse, or suicidal ideation were more likely to have PTSD (OR = 1.51 [1.42, 1.60], OR = 2.01 [1.89, 2.14], OR = 2.49 [2.26, 2.74], respectively). Table 5 provides a detailed overview of the multivariate binomial logistic regression model.

## Discussion

In this study, we provide an overview of the psychological status of the Libyan population during the COVID-19 outbreak and civil war in Libya. To our knowledge, this is the first study to report the prevalence of mental disorders, such as depression, anxiety, and PTSD, among the Libyan population with an adequate sample size that covered all major cities and regions of Libya. The study showed a higher prevalence of severe depressive symptoms (13.6%), moderate prevalence of severe anxiety (5.6%), and high levels of PTSD (19.8%) among the study population. There was a significant association between depressive symptoms, anxiety symptoms, and PTSD in most of the study variables. Young age, being female, unmarried, educated, or victims of domestic violence or abuse, work suspension during the pandemic, and having increased workload, financial issues, suicidal thoughts, or a family member with or hospitalized due to COVID-19 were significantly associated with a high likelihood of depressive and anxiety symptoms, as well as PTSD. These findings provide an overview of the mental health status of the Libyan population in the current situation of lockdown and civil war.

Several factors may have played a role in these results. The COVID-19 outbreak has affected several billion people worldwide. People have been restricted to their homes without their daily work and study activities, resulting in emotional tension and increased financial difficulties, especially for those with low wage incomes or those who work in small companies or working sectors.

During the study period, the civil war in Libya did not stop despite calls for a cease fire. The ongoing conflicts have resulted in high rates of internal displacement, with people having to leave their homes and belongings in attempts to stay away from the conflict areas that are exposed to the risk of theft, murder, and kidnapping. This has added to the stress of those people who have left their homes or are still living in conflict areas as displayed by the study, with approximately 1,256 (4%) of the study population reporting that they have left their homes and are internally displaced. In addition, internal displacement leads to an increase in financial difficulties as people have to pay for rent and other expenses, which has worsened the current financial crisis of Libya. No urgent plans or initiatives have been announced by the government in response to this ongoing crisis. In another study, 14,254 (45.2%) individuals of the study population reporting that they were suffering from a financial crisis. This may affect their mental health, as financial crises and recessions have been shown to have significant effects on mental health outcomes [18].

Several previous studies have discussed the effect of the COVID-19 pandemic on the mental health of populations. A study conducted in Italy on 18,147 respondents during the COVID-19 pandemic and lockdown [13] reported a depression rate of 17.3% compared to 13.6% in our study, an anxiety rate of 7.3% compared to 5.6% in our study, and a PTSD rate of 37% compared to 19.8% in our study. In another population-based study in China [5] conducted in 194 cities with 1,210 respondents, approximately 16.5% of the study population reported depressive symptoms and 28.8% reported moderate to severe anxiety; however, both anxiety and depression were measured using the Depression, Anxiety and Stress Scale (DASS-21), which is not the same tool used in the present study. Furthermore, in the study from China, 8.1% of the study population was reported to have moderate to severe stress, while in our study, 19.8% of the study population was found to have PTSD using the same tool. Both studies confirmed the same findings that young women were at a higher risk of mental disorders. Our study found that females are at a higher risk of mental disorders, which is in line with a previous study that concluded that women are at a higher risk of mental disorders. In a nationwide survey of 52,730 respondents in China [19], approximately 35% had psychological distress, and young women had significantly higher psychological distress. Another study conducted in Spain [7] showed that depression was present in 18.7% of respondents, anxiety in 21.6% of participants, and PTSD in 15.8% of participants, which is similar to our findings. Nonetheless, based on our results, older individuals were associated with a lower risk of mental distress. In addition, the study conducted in Spain showed that females and individuals with symptoms of COVID-19 were associated with higher psychological distress, similar to our findings. Nevertheless, they used the condensed tools PHQ-2 and GAD-2 for measurements in a smaller sample size than in our study. In addition, the mean age was 64.85 years, with few representatives from younger age groups. The higher prevalence of anxiety and PTSD among women might be the reason for the higher rate of these disorders in women during COVID-19 [20]. Therefore, younger age groups might be at an increased risk of mental disorders, and there is a need for urgent interventions aimed at relieving related distress during COVID-19.

The measurement of mental health status during a civil war and pandemic is an important aspect of healthcare systems. Being internally displaced and at a higher risk during civil war are associated with several common mental disorders, according to previous studies [21, 22]. However, psychiatrists and mental health specialists face several challenges in their work during the COVID-19 outbreak [6, 23]. The current lockdown represents a mental stress challenge, as people are concerned about the undetermined duration of lockdown, financial status, fear of infection, and subsequent hospitalization, with risk of death or severe COVID-19. Therefore, psychosocial support programs should be implemented by the government to support people during lockdown, especially for those who are affected by the pandemic or are regarded as vulnerable populations. This should be done by providing financial support and conducting social programs aimed at providing help to those living in conflict areas, especially children and the elderly, who are in need of support to reduce the mental distress associated with this conflict and pandemic. Furthermore, targeted intervention and determined actions employed by the authorities are needed to relieve distress and provide relief to patients hospitalized with COVID-19 and their relatives.

The current study has several limitations. First, the study depended on paper and online survey methods, and the study population may not have been representative of the elderly population as intended, especially as it comprised less than 10% of the study population. Second, it is likely that some people may not have had access to the Internet to complete the online study, while others could not complete the paper survey owing to illiteracy. This is especially likely for individuals in the older age groups. Third, as this was a cross-sectional study, there were difficulties in drawing causative relationships. Fourth, the study demonstrates a confounding effect of civil war that may need further investigation and may have affected the study outcomes, especially in cases in which certain people were affected more than others. There is a need to further understand the factors associated with the effect of civil war on physical abuse and specific stressful events caused by the civil war. However, the study has several strengths including a large representative sample from all regions, detailed questions and surveys along with the use of comprehensive tools, and a new tool for measuring the impact of lockdown.

To our knowledge, this is the first study to analyze the psychological impact of the COVID-19 pandemic and civil war in Libya. It provides important data on the psychological impact of COVID-19 in the African region, where COVID-19 may threaten the population at an increased risk owing to the current fragile healthcare systems and lack of mental health support. The study showed higher rates of psychological distress among the Libyan population with high rates of depression, anxiety, and PTSD. These findings warrant future studies on the development of strategies and interventions aimed at reducing the mental disease burden on the Libyan population.

## Supporting information

S1 File Arabic version of the survey. (PDF)

S2 File English version of the survey. (PDF)

## References

[1] Huang C, Wang Y, Li X, Ren L, Zhao J, Hu Y, et al. Clinical features of patients infected with 2019 novel coronavirus in Wuhan, China. Lancet (London, England). 2020;395(10223):497–506. Epub 2020/01/28. doi: 10.1016/s0140-6736(20)30183-5 ; PubMed Central PMCID: PMC7159299. 31986264 PMC7159299

[2] Dong E, Du H, Gardner L. An interactive web-based dashboard to track COVID-19 in real time. The Lancet Infectious diseases. 2020;20(5):533–4. Epub 2020/02/23. doi: 10.1016/S1473-3099(20)30120-1 ; PubMed Central PMCID: PMC7159018. 32087114 PMC7159018

[3] Rauch SAM, Simon NM, Rothbaum BO. Rising Tide: Responding to the Mental Health Impact of the COVID-19 Pandemic. n/a(n/a). doi: 10.1002/da.23058 34690589 PMC8475908

[4] Tian F, Li H, Tian S, Yang J, Shao J, Tian C. Psychological symptoms of ordinary Chinese citizens based on SCL-90 during the level I emergency response to COVID-19. Psychiatry Res. 2020;288:112992. Epub 2020/04/18. doi: 10.1016/j.psychres.2020.112992 ; PubMed Central PMCID: PMC7151383. 32302816 PMC7151383

[5] Wang C, Pan R, Wan X, Tan Y, Xu L, Ho CS, et al. Immediate Psychological Responses and Associated Factors during the Initial Stage of the 2019 Coronavirus Disease (COVID-19) Epidemic among the General Population in China. International journal of environmental research and public health. 2020;17(5). Epub 2020/03/12. doi: 10.3390/ijerph17051729 ; PubMed Central PMCID: PMC7084952. 32155789 PMC7084952

[6] Rajkumar RP. COVID-19 and mental health: A review of the existing literature. Asian J Psychiatr. 2020;52:102066-. doi: 10.1016/j.ajp.2020.102066 . 32302935 PMC7151415

[7] González-Sanguino C, Ausín B, Castellanos MÁ, Saiz J, López-Gómez A, Ugidos C, et al. Mental health consequences during the initial stage of the 2020 Coronavirus pandemic (COVID-19) in Spain. Brain, behavior, and immunity. 2020:S0889-1591(20)30812-6. doi: 10.1016/j.bbi.2020.05.040 . 32405150 PMC7219372

[8] Charlson FJ, Steel Z, Degenhardt L, Chey T, Silove D, Marnane C, et al. Predicting the impact of the 2011 conflict in Libya on population mental health: PTSD and depression prevalence and mental health service requirements. PloS one. 2012;7(7):e40593. Epub 2012/07/19. doi: 10.1371/journal.pone.0040593 ; PubMed Central PMCID: PMC3396632. 22808201 PMC3396632

[9] Musisi S, Kinyanda E. Long-Term Impact of War, Civil War, and Persecution in Civilian Populations-Conflict and Post-Traumatic Stress in African Communities. Front Psychiatry. 2020;11:20-. doi: 10.3389/fpsyt.2020.00020 . 32158407 PMC7051938

[10] Okasha A, Karam E, Okasha T. Mental health services in the Arab world. World psychiatry: official journal of the World Psychiatric Association (WPA). 2012;11(1):52–4. Epub 2012/02/02. doi: 10.1016/j.wpsyc.2012.01.008 ; PubMed Central PMCID: PMC3266748. 22295010 PMC3266748

[11] Rhouma AH, Husain N, Gire N, Chaudhry IB. Mental health services in Libya. BJPsych Int. 2016;13(3):70–1. doi: 10.1192/s2056474000001288 . 29093908 PMC5618881

[12] Abuazza A. The Arab Spring movement: a catalyst for reform at the psychiatric hospital in Tripoli, Libya. Int Psychiatry. 2013;10(3):56–8. . 31507734 PMC6735120

[13] Rossi R, Socci V, Talevi D, Mensi S, Niolu C, Pacitti F, et al. COVID-19 pandemic and lockdown measures impact on mental health among the general population in Italy. An N = 18147 web-based survey2020. 10.3389/fpsyt.2020.00790 PMC7426501 32848952

[14] Kroenke K, Spitzer RL, Williams JB. The PHQ-9: validity of a brief depression severity measure. Journal of general internal medicine. 2001;16(9):606–13. Epub 2001/09/15. doi: 10.1046/j.1525-1497.2001.016009606.x ; PubMed Central PMCID: PMC1495268. 11556941 PMC1495268

[15] Levis B, Benedetti A, Thombs BD. Accuracy of Patient Health Questionnaire-9 (PHQ-9) for screening to detect major depression: individual participant data meta-analysis. 2019;365:l1476. doi: 10.1136/bmj.l1476 %J BMJ PMC6454318 30967483

[16] Spitzer RL, Kroenke K, Williams JB, Löwe B. A brief measure for assessing generalized anxiety disorder: the GAD-7. Archives of internal medicine. 2006;166(10):1092–7. Epub 2006/05/24. doi: 10.1001/archinte.166.10.1092 . 16717171

[17] Beck JG, Grant DM, Read JP, Clapp JD, Coffey SF, Miller LM, et al. The impact of event scale-revised: psychometric properties in a sample of motor vehicle accident survivors. J Anxiety Disord. 2008;22(2):187–98. Epub 2007/02/24. doi: 10.1016/j.janxdis.2007.02.007 . 17369016 PMC2259224

[18] Hammarström A, Virtanen P. The importance of financial recession for mental health among students: short- and long-term analyses from an ecosocial perspective. J Public Health Res. 2019;8(2):1504-. doi: 10.4081/jphr.2019.1504 . 31579674 PMC6761466

[19] Qiu J, Shen B, Zhao M, Wang Z, Xie B, Xu Y. A nationwide survey of psychological distress among Chinese people in the COVID-19 epidemic: implications and policy recommendations. Gen Psychiatr. 2020;33(2):e100213-e. doi: 10.1136/gpsych-2020-100213 . 32215365 PMC7061893

[20] Haro JM, Palacín C, Vilagut G, Martínez M, Bernal M, Luque I, et al. [Prevalence of mental disorders and associated factors: results from the ESEMeD-Spain study]. Medicina clinica. 2006;126(12):445–51. Epub 2006/04/20. doi: 10.1157/13086324 . 16620730

[21] Siriwardhana C, Adikari A, Pannala G, Siribaddana S, Abas M, Sumathipala A, et al. Prolonged internal displacement and common mental disorders in Sri Lanka: the COMRAID study. PloS one. 2013;8(5):e64742-e. doi: 10.1371/journal.pone.0064742 . 23717656 PMC3661540

[22] Morina N, Akhtar A, Barth J, Schnyder U. Psychiatric Disorders in Refugees and Internally Displaced Persons After Forced Displacement: A Systematic Review. Front Psychiatry. 2018;9:433-. doi: 10.3389/fpsyt.2018.00433 . 30298022 PMC6160546

[23] Li W, Yang Y, Liu ZH, Zhao YJ, Zhang Q, Zhang L, et al. Progression of Mental Health Services during the COVID-19 Outbreak in China. International journal of biological sciences. 2020;16(10):1732–8. Epub 2020/04/01. doi: 10.7150/ijbs.45120 ; PubMed Central PMCID: PMC7098037. 32226291 PMC7098037

