# Supplementary material for: Impact of lockdown due to the COVID-19 pandemic on mental health among the Libyan population
Source: PLoS One. 2022 Apr 28;17(4):e0267426. doi: 10.1371/journal.pone.0267426 (PMC9049310; doi:10.1371/journal.pone.0267426)
Supplement: S1 File — (PDF) [file pone.0267426.s001.pdf]

استبيان حول مدي تأثرك بجائحة كورونا

إلي من يقوم بتعبئة الاستبيان ,

أخي الكريم / أختي الكريمة ... تحية طيبة

نتيجة إنتشار فايرس كورونا في الأونة الأخيرة و تأثيرها السلبي على الصحة النفسية على المجتمع , فإننا نطمح من خلال هذه الإستبيان لعمل دراسة احصائية تخص ليبيا تهدف الي كشف تأثير جائحة كورونا على كل من مواطن مقيم في ليبيا.

نحن مجموعة من الأطباء و الطلبة المتطوعين نسعى إلى تخفيف الأثار السلبية على مجتمعنا عن طريق معرفة مسببات و أثار جائحة كورونا على الصحة النفسية في هذا الوقت و معرفة كيفية التقليل منها لمساعدة الناس.

نرجو منك تعبئة هذه البيانات والتي قد تستغرق 10 دقائق من وقتك , علما بأنه لن يتم إستخدام اي معلومات شخصية و جميع المعلومات ستكون مشفرة وسوف نسعي للحفاظ علي سرية المعلومات التي سيتم استخدامها للأغراض الاحصائية فقط ...
يمكنك الاجابة عن كل سؤال في الفقرات الموجودة عن طريق اختيار أحد الإجابات , لهذا نرجو منك إعطاء إجاباتك بكل مصداقية لاظهار مدي تأثرك بجائحة كورونا.

يعد إكمالك للاستبيان موافقة منك علي المشاركة في البحث

البيانات الشخصية

- **العمر :** ------------------------------------
- **الجنس :**
  - ☐ ذكر
  - ☐ أنثي
- **الجنسية :**
  - ☐ ليبي
  - ☐ غير ليبي ولكن مقيم في ليبيا من أكثر من خمس سنوات
  - ☐ غير ليبي مقيم في ليبيا منذ اقل خمس سنوات
- **الحالة الاجتماعية :**
  - ☐ عازب/ة
  - ☐ متزوج/ة
  - ☐ ملطق/ة
  - ☐ أرمل/ة
- **هل لديك أطفال ؟**
  - ☐ لا
  - ☐ نعم
- **المؤهل العلمي :**
  - ☐ شهادة ابتدائية
  - ☐ شهادة اعدادية
  - ☐ شهادة ثانوية
  - ☐ خريج جامعي / معهد عالي
  - ☐ دراسات عليا
  - ☐

معلومات تتعلق بالحجر الصحي

- **العزل** :
  - ☐ عزلت نفسي بناء علي تعليمات الهيئات لحكمية
  - ☐ أنا تحت العزل لأني تعرضت للعدوي (نتيجة التحليل موجبة) .أو أني تعرضت لشخص مصاب
  - ☐ أنا حاليا في المستشفى
  - ☐ لا أمارس أي نوع من العزل الشخصي

- **قبل بداية الحجر" كم ساعة كنت تقضي (تقريبيا) خارج المنزل أو مكان السكن كل يوم ؟**
  - ☐ 0-1 ساعة يوميا
  - ☐ 2-3 ساعات يوميا
  - ☐ 4-5 ساعات يوميا
  - ☐ 6-8 ساعات يوميا
  - ☐ أكثر من 8 ساعات يوميا
- **"خلال فترة الحجر" كم ساعة كنت تقضي (تقريبيا) خارج المنزل أو مكان السكن كل يوم ؟**
  - ☐ 0-1 ساعة يوميا
  - ☐ 2-3 ساعات يوميا
  - ☐ 4-5 ساعات يوميا
  - ☐ 6-8 ساعات يوميا
  - ☐ أكثر من 8 ساعات يوميا
- **خلال فترة الحجر , أشعر بتحسن في المزاج بعد القيام بما يلي (يمكن اختيار أكثر من إجابة)**
  - ☐ ممارسة التمارين الرياضية
  - ☐ مشاهدة الأفلام والمسلسلات
  - ☐ متابعة الأخبار
  - ☐ تنفيذ بعض المهام والاعمال
  - ☐ القيام بأعمال المنزل
  - ☐ التحدث مع شخص
  - ☐ الابتعاد عن أشخاص مزعجين أقضي معهم فترة الحجر
  - ☐ تناول الطعام مع العائلة
- **خلال فترة الحجر , هل تواصلت مع أي شخص لا يقيم معك في نفس المسكن عن طريق (يمكن اختيار أكثر من إجابة)**
  - ☐ مقابلته وجها لوجه
  - ☐ التحدث بالهاتف
  - ☐ المحادثة المباشرة عبر الانترنت
  - ☐ محادثة فيديو مباشرة عبر الانترنت
  - ☐ التحدث عن طريق الرسائل والدردشة
- **هل تعتقد أن الحجر الصحي يعتبر فكرة سديدة؟**
  - ☐ نعم
  - ☐ لا
- **كيف كان مستوي التوتر قبل تطبيق قرار الحجر الصحي ؟**
  - ☐ كنت هادئا
  - ☐ لم أكن هادئا أو متوترا
  - ☐ كنت متوترا
  - ☐ كنت متوترا جدا
- **ما مستوي التوتر الذي تتشعر به الأن ؟**
  - ☐ أنا هادئ
  - ☐ لست هادئا أو متوترا
  - ☐ أنا متوتر
  - ☐ أنا متوترا جدا
- **أتلقي معلومات عن انتشار الوباء عن طريق.......... (يمكن اختيار أكثر من إجابة)**
  - ☐ بيانات رسمية صادرة عن السلطات الحكومية
  - ☐ وسائل الاعلام
  - ☐ الأصدقاء, العائلة والجيران
  - ☐ وسائل التواصل الاجتماعي
  - ☐ لا أتلقي أي معلومات عن انتشار الوباء

- **منذ بداية جائحة كورونا :**
  - ☐ لم تظهر علي أعراض العدوي ولم أتعرض لأي شخص يحمل العدوي
  - ☐ تعرضت لأشخاص مصابين بالعدوي, ولكنني لم أصب بالعدوي
  - ☐ تعرضت للعدوي, ولكنني لم أدخل المستشفى
  - ☐ دخلت المستشفى بسبب إصابتي بالعدوي
- **وضع العمل بعد جائحة كورونا**
  - ☐ لم يتغير وضع العمل علي ما كان عليه من قبل
  - ☐ ازداد الحمل الوظيفي علي ما كان عليه
  - ☐ تغيرت طريقة العمل (عمل عن بعد علي سبيل المثال)
  - ☐ أنا متوقف عن العمل
- **هل أصيب أحد أفراد أسرتك أو شخص عزيز عليك بوباء كورونا**
  - ☐ لا
  - ☐ نعم, أصيب بالعدوي
  - ☐ نعم, تعرض للعدوي وأدخل المستشفى
  - ☐ نعم, توفي بسبب العدوي
- **هل عانيت من أي من المشاكل الأتية خلال فترة الحجر؟** (يمكن اختيار أكثر من إجابة)
  - ☐ الاكتئاب
  - ☐ مشاكل عائلية
  - ☐ مشاكل عاطفية
  - ☐ مشاكل مالية
  - ☐ فترات من القلق والتوتر
  - ☐ فكرت جديا في الانتحار
- **ما مستوي تقيدك بشروط العزل والمسافة الأمنة التي فرضتها السلطات لمواجهة جائحة كورونا**
  - ☐ لم ألتزم بشروط العزل
  - ☐ التزمت قليلا
  - ☐ التزمت قدر المستطاع
  - ☐ التزمت أغلب الأوقات
  - ☐ لم أخالف شروط العزل مطلقا

معلومات تتعلق بالصحة النفسية

كم عانيت من المشاكل التالية خلال الشهر الماضي (ضع علامة √ أمام الإجابة الأنسب)	أبدا	بعض الأيام	أغلب الأيام	كل الأيام تقريبا
قلة الاهتمام أو الرغبة في ممارسة أي نشاط أو عمل	☐	☐	☐	☐
الشعور بالحزن أو ضيق الصدر أو اليأس	☐	☐	☐	☐
صعوبة في الاستسلام للنوم أو الانتظام في النوم أو النوم أكثر من المعتاد	☐	☐	☐	☐
الشعور بالتعب أو الفشل العام	☐	☐	☐	☐
قلة الشهية أو الافراط في الأكل	☐	☐	☐	☐
الشعور بعدم الرضا عن النفس أو الفشل أو الإحباط	☐	☐	☐	☐
صعوبة في التركيز عند القيام بشيء , مثل المطالعة أو الدراسة أو مشاهدة التلفاز	☐	☐	☐	☐
بطء في الحركة أو الكلام بدرجة ملحوظة من الأخرين, أو عكس ذلك بكثرة الحركة وعدم البقاء ساكنا	☐	☐	☐	☐
الشعور باليأس من الحياة وتمني الموت أو التفكير في إيذاء نفسك	☐	☐	☐	☐
قمت بإيذاء نفسي أو تسببت في تعريض نفسي للخطر	☐	☐	☐	☐
خططت للقيام بالانتحار	☐	☐	☐	☐

كم عانيت من المشاكل التالية خلال الشهر الماضي (ضع علامة √ أمام الإجابة الأنسب)	أبدا	بعض الأيام	أغلب الأيام	كل الأيام تقريبا
الشعور بالغضب أو القلق أو الانفعال الشديد	☐	☐	☐	☐
عدم القدرة على إنهاء القلق أو التحكّم فيه	☐	☐	☐	☐
القلق المفرط على أشياء مختلفة	☐	☐	☐	☐
الصعوبة في الاسترخاء	☐	☐	☐	☐
شدة الاضطراب لدرجة صعوبة البقاء ساكنا	☐	☐	☐	☐
السرعة في الانزعاج أو الانفعال	☐	☐	☐	☐
الشّعور بالخوف كما لو أن شيئا سيئا قد يحدث	☐	☐	☐	☐

هل شعرت بفقدان الرغبة في العمل	☐ نعم	☐ لا		
هل واجهتك مشاكل في الاستسلام للنوم أو البقاء نائما؟	☐ نعم	☐ لا		
هل حاولت التخلص من القلق عن طريق تعاطي الدخان, الكحول, العقاقير أو المخدرات؟	☐ نعم	☐ لا		
هل افتقدت وجود الأشخاص المقربين الذين يمكنك الاعتماد عليهم عند الحاجة؟ (للدعم النفسي, للاهتمام بالأطفال, لإيصالك إلي المستشفى أو السوق التجاري, للاهتمام بك حال مرضك الخ......)	☐ نعم	☐ لا		
هل سبق أن تلقيت علاجا خاصا بالمشاكل النفسية؟ (الاكتئاب, القلق الخ.....)	☐	☐		
إذا قمت بتحديد أي مما سبق إلي أي مدي منعتك هذه المشاكل من القيام بعملك أو الاعتناء بشئونك الشخصية أو التعامل مع الأخرين	☐ لا أجد مشقة	☐ بعض المشقة	☐ صعوبة ملحوظة	☐ صعب جدا

	موقف أو حادثة تعرضت لها أثناء فترة الحرب أثر علي صحتك النفسية	اختر الخانة الأنسب (√)				
		لا أبدا	قليلا	بشكل ملحوظ	كثيرا	كثيرا جدا
	أدني تذكير بالموقف يعيد لذاكرتك مشاعر متعلقة بالحادثة					
	مواقف أخري ذكرتني بالحادثة					
	شعرت بالانفعال والغضب					
	حاولت منع نفسي من الشعور بالاكتئاب عند التفكير بالحادثة أو عند تذكيري بها					
	أجد نفسي أفكر في الحادثة دون قصد					
	أشعر بأن الحادثة لم تكن حقيقية أو كأنها لم تحدث					
	حاولت الابتعاد عن كل ما يذكرني بالحادثة					
	صور الحادثة تظهر في ذهني					
	كنت متوترا وسريع التأثر					
	حاولت عدم التفكير بالحادثة					
	أعي جيدا وجود مشاعر مكبوتة تتعلق بالحادثة, ولكني لم أتعامل معها كما يجب					
	أشعر بفقدان في الاحساس فيما يتعلق بالحادثة					
	أشعر وأتصرف كما لو أنني لا زلت أعيش في زمن الحادثة					
	عانيت صعوبة في النوم					
	عانيت صعوبة في البقاء نائما					
	اجتاحتني مشاعر قوية يصعب كبتها بسبب الحادثة					
	حاولت إزالة الحادثة من ذاكرتي					
	عانيت من صعوبة في التركيز					
	أي تذكير بالحادثة سبب لي أعراض ظاهرة , مثل التعرق المفرط, صعوبة التنفس, الغثيان أو تسارع نبضات القلب					
	راودتني أحلام تتعلق بالحادثة					
	أحسست بأنني دائما مترقب ومحترز					
	حاولت أن لا أتحدث عن الحادثة					

نشكر لكم مشاركتكم في هذا الاستبيان
