# Supplementary material for: Impact of lockdown due to the COVID-19 pandemic on mental health among the Libyan population
Source: PLoS One. 2022 Apr 28;17(4):e0267426. doi: 10.1371/journal.pone.0267426 (PMC9049310; doi:10.1371/journal.pone.0267426)
Supplement: S2 File — (PDF) [file pone.0267426.s002.pdf]

To those who wish to participate in this questionnaire,

Dear Sir / Madam

Greetings……

In light of the recent spread of the COVID-19 Pandemic and the negative impact it had on the mental health of our community, we aspire through this questionnaire to conduct a statistical study in Libya which aims to reveal the psychological impact of the COVID pandemic on the Libyan Population.

We are a group of doctors and volunteer students seeking to reduce the negative impact of this pandemic on the psychological well-being of our society by identifying the possible effects on mental health at this time and attempting to reduce their impact on our community .

We ask you to fill out this form, which may take about 10 minutes of your time, knowing that no personal information is required and all the data collected will be used for statistical purposes only, your data shall be encrypted and confidentiality of your information shall be maintained at all times.

You may answer all the questions below by choosing one of the answers available, we ask you to register your responses with absolute sincerity I order to illustrate how affected you are by the Corona pandemic.

Completion of the questionnaire is considered as consent to participate in this research

Personal data

Age: ------------------------------------

Gender:

- ☐ Male
- ☐ Female

Nationality:

- ☐ Libyan
- ☐ Non-Libyan, but has resided in Libya for more than five years
- ☐ A non-Libyan residing in Libya for less than five years

Marital status:

- ☐ Single
- ☐ Married / married

- ☐ Divorced
- ☐ Widower

Do you have children?

- ☐ No.
- ☐ Yes

Highest Qualification:

- ☐ Primary certificate
- ☐ Preparatory Certificate
- ☐ Secondary school certificate
- ☐ University graduate / higher institute
- ☐ Postgraduate studies

Quarantine information

Isolation**:**

- ☐ I isolated myself according to the instructions of Government Authorities
- ☐ I am under isolation because I have been infected (positive test result). Or I have been exposed to an infected person
- ☐ I am currently hospitalized as a result of infection
- ☐ I do not practice any kind of personal isolation

Before the start of the Lockdown, "How many hours did you spend (approximately) outside your home or residence every day?

- ☐ 1 - 0 hours per day
- ☐ 3 - 2 hours a day
- ☐ 5 - 4 hours a day
- ☐ 8 - 6 hours a day
- ☐ More than 8 hours a day

“During the Lockdown period” How many hours did you spend (approximately) outside your home or residence every day?

- ☐ 1 - 0 hours per day
- ☐ 3 - 2 hours a day
- ☐ 5 - 4 hours a day
- ☐ 8 - 6 hours a day
- ☐ More than 8 hours a day

During the Lockdown period, I feel a better after doing the following (more than one answer can be chosen)

- Physical activity & exercise
- Watch movies and series
- Follow the news
- Carry out some tasks or assignments
- Doing housework
- Talk to someone
- Stay away from annoying people whom I spend the lockdown with
- Eating with my family

During the lockdown period, did you contact any person who does not reside with you in the same residence through (you can choose more than one answer)

- Meeting him face to face
- Speak on the phone
- Live chat online
- Live video chat online
- chat and text messages

Do you think quarantine is a good idea?

- Yes
- No.

How was the tension level before the lockdown was implemented?

- I was calm
- I was not calm or tense
- I was so nervous
- I was so nervous

What is the tension level during lockdown?

- I am calm
- Not calm or tense
- I'm tense
- I'm very nervous

I receive information about the spread of the epidemic through .... (More than one answer can be selected)

- Official reports issued by government authorities
- The media
- Friends, family and neighbors
- Social media
- I am not receiving any information on the spread of the epidemic

Since the beginning of the Corona pandemic:

- ☐ I had no symptoms of infection, nor was I exposed to anyone with infection
- ☐ I was exposed to people with infection, but I did not develop any symptoms
- ☐ I was infected, but I was not hospitalized
- ☐ I was hospitalized because of my infection

Work status after the Corona pandemic

- ☐ The work situation has not changed
- ☐ The work load has increased
- ☐ The modus operandi changed (remote work, for example)
- ☐ I am not currently working

Has someone in your family or someone close to you been infected with the COVID-19 virus?

- ☐ No.
- ☐ Yes, he was infected
- ☐ Yes, he was infected and admitted to hospital
- ☐ Yes, he died due to an infection

Have you had any of the following problems during the lockdown period? (More than one answer can be selected)

- ☐ Depression
- ☐ Family problems
- ☐ Emotional problems
- ☐ Financial problems
- ☐ periods of anxiety and stress
- ☐ Seriously considered suicide

How would you describe your level of compliance with the conditions of isolation and the safe distance imposed by the government authorities to face the Corona pandemic ?

- ☐ I did not adhere to the isolation instructions
- ☐ a little
- ☐ as much as possible
- ☐ most of the time
- ☐ I have never violated the terms of isolation

Mental Health

Which of the following problems have you experienced during the past month (tick the most appropriate answer)	Not at all	Several days	More than half the days	Nearly everyday
1. Little interest or pleasure in doing things	0	1	2	3
2. Feeling down, depressed, or helpless	0	1	2	3
3. Trouble falling or staying asleep, or sleeping too much	0	1	2	3
4. Feeling tired or having little energy	0	1	2	3
5. Poor appetite or overeating	0	1	2	3
6. Feeling bad about yourself – or that you are a failure or have let yourself or your family down	0	1	2	3
7. Trouble concentrating on things, such as reading the newspaper or watching television	0	1	2	3
8. Moving or speaking so slowly that other people could have noticed. Or the opposite_ being so fidgety or restless that you have been moving around a lot more than usual	0	1	2	3
9. Thoughts that you would be better off dead, or of hurting yourself	0	1	2	3
10. I hurt myself or I put myself in danger	0	1	2	3
11. I planned to commit suicide	0	1	2	3

How many of you have experienced the following problems during the past month (tick the most appropriate answer)			
Feeling nervous, anxious or on edge?			
☐ Not at all	☐ Several Days	☐ More than Half the days	☐ Nearly every day
Not being able to stop or control worrying?			
☐ Not at all	☐ Several Days	☐ More than Half the days	☐ Nearly every day
Worrying too much about different things?			
☐ Not at all	☐ Several Days	☐ More than Half the days	☐ Nearly every day
Trouble relaxing?			
☐ Not at all	☐ Several Days	☐ More than Half the days	☐ Nearly every day
Being so restless that it is hard to sit still?			
☐ Not at all	☐ Several Days	☐ More than Half the days	☐ Nearly every day
Becoming easily annoyed or irritable?			
☐ Not at all	☐ Several Days	☐ More than Half the days	☐ Nearly every day
Feeling afraid as if something awful might happen?			
☐ Not at all	☐ Several Days	☐ More than Half the days	☐ Nearly every day

Have you felt a loss of desire to work	☐ Yes	☐ No
Did you encounter problems surrendering to sleep or staying asleep?	☐ Yes	☐ No
Have you tried to eliminate anxiety by resorting to smoking cigarettes, using alcohol, or drugs?	☐ Yes	☐ No
Have you missed the presence of people you can depend on when needed? For psychological support, caring for children, to get you to the hospital or market, to take care of you if you are sick etc ...	☐ Yes	☐ No
Have you ever received a treatment for mental problems? Depression, anxiety, etc.	☐ Yes	☐ No

If you specify any of the above to what extent these problems prevent you from<br>Doing your work, looking after your personal affairs, or dealing with others	☐ None	☐ Some trouble	☐ Significantly	☐ Very difficult

The situation or incident you experienced during the war that affected your mental health	Not at all	A little	Moderately	Quite a bit	Extremely
1. Any reminder brought back feelings about it	0	1	2	3	4
2. I had trouble staying asleep	0	1	2	3	4
3. Other things kept making me think about it.	0	1	2	3	4
4. I felt irritable and angry	0	1	2	3	4
5. I avoided letting myself get upset when I thought about it or was reminded of it	0	1	2	3	4
6. I thought about it when I didn't mean to	0	1	2	3	4
7. I felt as if it hadn't happened or wasn't real.	0	1	2	3	4
8. I stayed away from reminders of it.	0	1	2	3	4
9. Pictures about it popped into my mind.	0	1	2	3	4
10. I was jumpy and easily startled.	0	1	2	3	4
11. I tried not to think about it.	0	1	2	3	4
12. I was aware that I still had a lot of feelings about it, but I didn't deal with them.	0	1	2	3	4
13. My feelings about it were kind of numb.	0	1	2	3	4
14. I found myself acting or feeling like I was back at that time.	0	1	2	3	4
15. I had trouble falling asleep.	0	1	2	3	4
16. I had waves of strong feelings about it.	0	1	2	3	4
17. I tried to remove it from my memory.	0	1	2	3	4
18. I had trouble concentrating.	0	1	2	3	4
19. Reminders of it caused me to have physical reactions, such as sweating, trouble breathing, nausea, or a pounding heart.	0	1	2	3	4
20. I had dreams about it.	0	1	2	3	4
21. I felt watchful and on-guard.	0	1	2	3	4
22. I tried not to talk about it.	0	1	2	3	4
